# *Listeria monocytogenes* endophthalmitis – case report and review of risk factors and treatment outcomes

**DOI:** 10.1186/s12879-016-1680-2

**Published:** 2016-07-16

**Authors:** Anna Bajor, Anke Luhr, Dorothee Brockmann, Sebastian Suerbaum, Carsten Framme, Ludwig Sedlacek

**Affiliations:** University Eye Hospital, Hannover Medical School, Carl-Neuberg-Str. 1, D-30625 Hannover, Germany; Institute for Medical Microbiology and Hospital Epidemiology, Hannover Medical School, Hannover, Germany

**Keywords:** Endophthalmitis, Endogenous, Listeria monocytogenes, Uveitis, Dark hypopyon

## Abstract

**Background:**

The majority of cases of endophthalmitis are caused by exogenous pathogens; only 5–10 % are of endogenous origin. One cause of these rare cases of endogenous endophthalmitis is *Listeria monocytogenes*. Twenty-six cases of endophthalmitis due to this pathogen have been published over the last twenty years. The aim of this review is to summarize the main risk factors and common clinical findings of endogenous endophthalmitis due to *Listeria monocytogenes*.

**Case presentation:**

We report on a 62-year-old female presenting with a sterile hypopyon iritis with secondary glaucoma and an underlying rheumatoid disease. In microbiological analysis we identified *Listeria monocytogenes*. Further we searched through all published cases for typical signs, risk factors, details of medical and surgical treatment and outcome of endogenous endophthalmitis due to this rare pathogen.

Ocular symptoms in almost all of these published cases included pain, redness of the eye, and decreased vision. Main clinical features included elevated intraocular pressure and fibrinous anterior chamber reaction, as well as a dark hypopyon. While the infection is typically spread endogenously, neither an exogenous nor endogenous source of infection could be identified in most cases. Immunocompromised patients are at higher risk of being infected than immunocompetent patients. The clinical course of endophthalmitis caused by *Listeria monocytogenes* had different visual outcomes. In some cases, the infection led to enucleation, blindness, or strong visual loss, whereas most patients showed a tendency of visual improvement during therapy.

**Conclusion:**

Early diagnosis and treatment initiation are crucial factors in the outcome of endogenous endophthalmitis caused by *Listeria monocytogenes*. This possible differential diagnosis should be kept in mind while treating patients with presumable sterile hypopyon and anterior uveitis having a high intraocular pressure. A bacterial source should be considered with a prompt initiation of systemic antibiotic treatment, mainly in immunocompromised patients, who develop endogenous anterior uveitis. An appropriate microbiological sampling is essential to detect atypical microorganisms and to choose an effective antibiotic treatment.

## Background

Endophthalmitis is a serious intraocular inflammatory disorder affecting the vitreous cavity that can occur after ocular surgery, trauma, or as a consequence of systemic infection [1]. It is one of the most devastating diagnoses in ophthalmology and often leads to loss of vision. Therefore, an immediate and effective treatment is necessary for satisfactory visual results. The most common form of endophthalmitis is caused exogenously following ocular surgery (67 %) [2, 3]. About 25 % of endophthalmitis cases are a result of ocular trauma [4]. Endogenous endophthalmitis accounts for only 5–10 % of all endophthalmitis cases. This occurs, when microorganisms from the bloodstream reach the eye, cross the blood-retina barrier, and infect ocular tissue [3, 5–7]. Risk factors include immunocompromised patient, diabetes mellitus, HIV infection, cancer, renal failure requiring haemodialysis, cardiac disease, steroid therapy, and indwelling intravenous catheters [4–7]. The causative pathogen can be successfully identified in more than 75 % of cases of endogenous endophthalmitis via intraocular specimen, blood culture, cerebrospinal fluid, or urine [4, 5, 8]. The pathogen *Listeria monocytogenes* is a very rare cause of endogenous endophthalmitis. There have been only twenty-six published cases since Goodner and Okumoto in 1967 [9–34].

## Case presentation

A 62-year-old woman presented to our emergency department with pain, redness, and decreased vision in the left eye, as well as nausea and vomiting. She reported having a “flu like” illness for three days prior to the referral, without neck stiffness and fever. Anamnestic the left eye showed initial symptoms of redness and a slightly blurred vision only two days before. There was no history of previous trauma, infection, or surgery. Her medical history was notable for diabetes mellitus type II, chronic rheumatoid arthritis, spondylitis ankylosans (mainly thoracic), HIT syndrome, thyroid disease, and obesity. Her regular medication at home consisted of a systemic therapy with levothyroxine/potassium iodide 50 μg/100 μg 1-0-0 for thyroid disease, metformin 1 g 1-0-1 and insulin (Novo Rapid, Levemir) to adjust diabetes mellitus and methotrexate 15 mg with folic acid once a week, as well as adalimumab 40 mg subcutaneous every 14 days for rheumatoid arthritis. She took additionally the medication morphine sulphate 30 mg 1-0-1 for chronic pain from rheumatoid arthritis and spondylitis ankylosans.

**Fig. 1:**
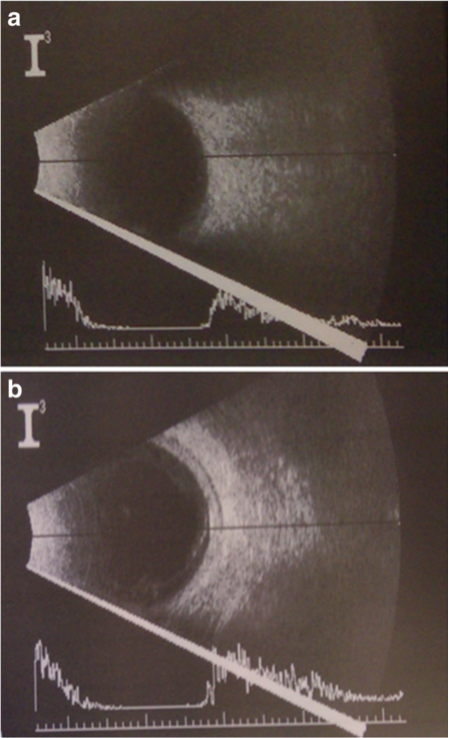
Ultrasonography (B-scan), left eye, day 3. **a** First look at 8.00 am; vitreous with no evidence of infiltration, no retinal detachment. **b** Second look at 2.00 pm; vitreous appears condensed and infiltrated at the posterior hyaloid membrane compared to the first look of the day, no retinal detachment

In clinical examination upon admission, the patient denied headache and did not show signs of meningitis or sinus thrombosis. In the ophthalmological examination, the VA of the left eye was reduced to 0.5 without refractive correction (sc) and the initial intraocular pressure was elevated to 48 mmHg. Our examination of the left eye revealed an injected conjunctiva and microcystic corneal edema. The anterior chamber contained a fibrinous inflammatory reaction in the pupillary level with a white hypopyon and hyperemia of the iris. Therefore, neither the lens nor the ocular fundus could be clearly visualized. An ultrasound scan of the eye revealed no evidence of vitreous debris, retinal detachment, or intraocular foreign body (Fig. 1a).

The right eye was asymptomatic and showed no noticeable diagnostic findings, aside from age-appropriate mild changes of the lens. The VA on the right eye was 0.7 sc and ocular pressure was 11 mmHg. The cover test revealed an orthophoric status with normal ocular motility. The vital parameters were in normal range, with the blood pressure of 140/80 mmHg, pulse 68/minute and a temperature of 36.6° Celsius.

Our initial diagnosis was an anterior hypertensive uveitis of the left eye, considering the history of spondylitis ankylosans. The elevated intraocular pressure as well as the hypopyon, were inconsistent with the differential diagnosis of anterior uveitis in the context of rheumatoid arthritis. Treatment with local and systemic corticosteroids was initiated. Topical brinzolamide (10 mg/ml, twice per day), timolol (0,5 %, twice per day) and brimonidine (2 mg/ml three times per day), as well as oral acetazolamide 250 mg daily, were administered for intraocular pressure control.

No significant improvement was seen the following day (day 2). Differential diagnoses like endogenous endophthalmitis and a neoplastic process were taken into consideration. Treatment with ciprofloxacin (400 mg intravenous twice a day), due to patients reported history of penicillin allergy, and topical kanamycin (5 mg/ml) and ofloxacin (3 mg/ml) half-hourly was initiated. Towards the evening of the same day (day 2), an increased hypopyon from 1.5 mm to 3 mm was noted, reinforcing our suspicion of an endogenous endophthalmitis. Anterior chamber tap was performed and examined for microbial pathogens by Gram stain, culturing, and histopathology. No signs for bacterial or fungal infections were seen in microscopy. Local therapy was initiated in the anterior chamber with gentamicin 0.1 ml (1.0 mg), vancomycin 0.1 ml (1.0 mg) and dexamethasone 0.1 ml (1.0 mg). By this time, the intraocular pressure decreased to an acceptable level of 21 mmHg, but VA was reduced to hand movements vision. Due to the progressive course of inflammation seen by increasing hypopyon and beginning vitreous opacities assessed by ultrasound scan (Fig. 1b), a pars plana vitrectomy was performed (on day 3). During the surgical procedure a moderate infiltration of the vitreous could be visualized without any signs of retina involvement.

The following day, 48 h hours after anterior chamber irrigation (day 4), the microbiological culture of the anterior chamber tap showed an infection with *Listeria monocytogenes*. The recommended first line therapy for *Listeria monocytogenes* ampicillin often combined with gentamycin could not be administered due to known allergy to penicillin. Instead, a treatment with meropenem 1 g t.i.d. and gentamycin 80 mg t.i.d. intravenously was initiated.

No gastroenteritic infection was mentioned by the patient in her (or in her household’s) medical history. Results from laboratory examinations were unremarkable and blood cultures were negative in microbiological examination. Chest X ray and transthoracic echocardiography showed no sign for a pathological process. Computer tomography of skull and orbita showed signs of ethmoidal sinusitis without evidence of bony erosions. Retrobulbar soft tissue and the cerebrum were unremarkable, and no signs of brain abscess could be identified. The ability to assess the abdominal ultrasound scan was significantly restricted due to flatulence and obesity. The ultrasound revealed hepatosplenomegaly and a status after cholecystectomy. No free liquid or any other evidence of colitis in the abdomen could be seen. A magnetic resonance imaging was refused by the patient due to claustrophobia, as was a lumbar puncture for further diagnosis. An extensive systemic evaluation failed to identify the source of *Listeria monocytogenes* infection. Additionally, there were no unusual incidences of infections noted in our clinic or in the city during this time period.

During this anti-infective treatment, and after the previous performed anterior chamber tap irrigation and pars plana vitrectomy, hypopyon did not reoccur. The inflammatory cells in the anterior chamber still persisted. Further the intraocular pressure increased to values over 35 mmHg, which required another lavage and irrigation with gentamicin, vancomycin and dexamethasone of the anterior chamber (on day 10). The clinical situation stabilized after this intervention and the inflammation of the anterior chamber slowly resolved. However, VA was limited and unsteady between 0.05 sc and 0.16 sc due to a severe corneal edema and a cataract that had developed in the patient’s eye. Patient signed out against medical advice after nineteen days of hospitalization. The VA at that time was on the left eye 0.1 sc and the intraocular pressure 10 mmHg. The eye showed conjunctival injection, the cornea had a 5.6 × 3.9 mm epithelial defect, descemet folds, and endothelial pigmentation. The anterior chamber was deep without inflammatory cells and the pupil showed posterior synechiae. The ultrasound scan showed a circumferentially attached retina and no signs for vitreous debris. Patient left with current topical steroid and antibiotic therapy consisting of prednisolonacetat (10 mg/ml) 8×/day, kanamycin (5 mg/ml), and ofloxacin (3 mg/ml) each 5×/day, as well as systemic corticosteroids 20 mg once daily.

**Fig. 2:**
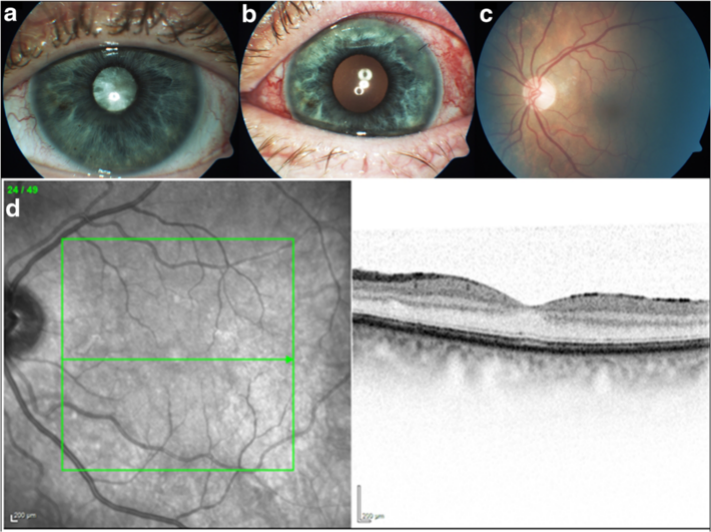
Complicated cataract with elevated intraocular pressure. Slit lamp examination of the left eye showing a dense cataract with no visualization of the fundus (**a**). Three days after performing cataract surgery (**b**, **c**). Optical coherence tomography of the macula (**d**)

Twenty-two weeks later the patient consulted our ward again with symptoms of pressure on the left eye and enhanced epiphora. The VA was reduced to hand movements vision and the IOP was 33 mmHg. The slit lamp examination of the left eye revealed a corneal edema, central endothelial precipitates and a circular injected conjunctiva. The anterior chamber was completely free of irritation without a hypopyon or other signs for an anterior uveitis. Due to a dense cataract the fundus could not be visualized (Fig. 2a). An ultrasound scan of the eye revealed no evidence of vitreous debris or signs of endophthalmitis. In the absence of inflammatory signs, we assumed an increasing cataract with progressive narrowing of the chamber angle to be responsible for the elevated intraocular pressure. A phacoemulsification of the lens was performed, followed by an implantation of an intraocular lens. The VA improved after the operation to 0.5 sc, while the intraocular pressure remained normotensive. The fundus showed unremarkable findings with normal configuration of retinal macula layers shown in an optical coherence examination (Fig. 2b, c, d). An autoimmune component source could not be excluded.

Five weeks later the patient complained about severe pain of the left eye, nausea, and vomiting. We saw a decompensation of the intraocular pressure on the left eye up to 58 mmHg. VA dropped to counting fingers. Due to a massive corneal edema, any further examination of the eye was not possible. After reduction of eye pressure and clearing of cornea, a hyperemic iris and cellular infiltration of the anterior chamber could be noted. There was no infiltration of the retina or vitreous. To exclude a relapse of endophthalmitis, samples of the anterior chamber and blood cultures for microbiological examination were taken. So far there was the possibility of a relapse of the endophthalmitis with *Listeria monocytogenes*. For this reason an antibiotic therapy with meropenem, gentamicin and a systemic steroid therapy (80 mg once daily) was initiated. An investigation of potential origin of infection was taken up again, without any positive results (chest x-ray, urine status, abdominal ultrasound scan, and differential blood count). The sample of the anterior chamber was sterile in microbiological examination, and a PCR for herpes simplex virus was negative. The blood cultures showed *Staphylococcus capitis*, most likely in the context of contamination, since the patient had no systemic signs of infection or endocarditis. Intravenous therapy was continued for thirteen days, followed by oral therapy with minocyclin 150 mg twice a day for four weeks.

Last clinical examination of the patient, twenty-seven months after first consultation, showed no inflammation of anterior chamber or vitreous of the left eye. The VA on the left eye was 0.7 with the best correction and the intraocular pressure was 10 mmHg.

Today (after 35 months) the patient reported continued use of local therapy with corticosteroids three times a day. Any attempted withdrawal trials led to renewed opacities and blurred vision. With this single treatment without any antibiotics, there have been no further ophthalmologic problems or other signs for a systemic infectious disease. Therefore, we assume a complete healing of the bacterial infection, but a lasting chronic uveitis.

## Review on symptoms, clinic, treatment and outcome of published cases

We reviewed all published cases describing endophthalmitis caused by *Listeria monocytogenes* between 1967 and May 2014 via research on PubMed. The search was performed using the keywords “Listeria”, “endophthalmitis” and “endogenous”. Only articles written in English and German are reviewed, although some key parameters from previously reviewed articles originally written in French were included.

Ocular symptoms in almost all cases included pain (20/23; 87 %), redness of the eye (15/23; 65 %) and decreased vision (13/23; 56 %). The only other described symptom was photophobia (3/23; 13 %), which occurred relatively seldom as it is a common symptom of uveitis. The clinical features included elevated intraocular pressure and fibrinous anterior chamber reaction (Table 1). Several patients developed a dark hypopyon (23/27; 85 %). The median age of the patients was 62 (interquartile range (IQR) =18) years, with the youngest being 24 and the oldest at 88 years (Table 1, 2). The present case, as well as nine of the twenty-six published cases of *L. monocytogenes* endophthalmitis, occurred in immunocompromised patients (37 %) [11, 14, 17, 19, 22, 25, 27, 29, 34]. Five cases (19 %) occurred in patients with chronic diseases such as neurodermitis, sacroiliitis, diabetes mellitus (present case), arterial hypertension, hypothyroidism, or previous history of cancer or surgery (coronary artery bypass graft) [16, 21, 24, 26, 30] (Table 2) . Six cases (22 %) occurred in elderly patients (age 62–76) in good physical health [9, 10, 12, 13, 18, 23, 33], and the remaining four cases (15 %) affected young (age 27–47), healthy individuals [20, 28, 31, 32]. More specifically, the recently published cases describe two young patients (age 27, 28), both having a history of refractive ocular surgery [31, 32]. In both of these cases, an exogenous infection is more apparent, whereas in all other reported cases an exogenous source of infection could not be identified.

**Table 1:** Onset of ocular symptoms

Case	Age/Sex	IOP (in mmHg)	Ocular symptoms	Fibrin reaction	Keratic precipitates	Hypopyon	Initial diagnosis
1 [9]	76 M	43	Red, uncomfortable, irritation, pain, poor vision	+	+		Acute anterior uveitis
2 [10]	62 M	44	Asymptomatic redness, decreased vision the following day	+		+	Anterior uveitis and corneal edema
3 [11]	69 M	39	Redness, pain	+			Acute iritis with secondary glaucoma
4 [12]	68 M	68	Redness, photophobia, pain	+	+	+	Anterior uveitis and corneal edema
5 [13]	68 F	52	Sudden onset of pain, decreased visual acuity	+		+	Necrotic ciliary body melanoma DD glaucomacyclitic crisis
6 [14]	57 F	30	Sudden onset of pain one week before	+	+	+	
7 [15]	49 F	46		+		+	
8 [16]	63 M	45	Pain, photophobia, decreased vision	+	+	+	Acute anterior hypertensive uveitis
9 [17]	50 F	40		+		+	
10 [18]	75 M	32	Pain, redness 2 days prior	+	+	+	
11 [19]	52 M	50	Itching, redness and pain			+	
12 [34]	76 F	44		+		+	
13 [20]	47 F	42	Pain, 6 days prior		+	+	Anterior hypertensive uveitis
14 [21]	67 F	42	Pain, redness, decreased vision				
15 [22]	55 M	50	Decreased vision and pain of the eye	+	+	+	
16 [23]	73 M	37	Redness, pain, blurred vision since 2 days	+	+	+	Granulomatoes anterior uveitis
17 [24]	51 F	44	Irritation, pain, decreased vision		+	+	
18 [25]	62 M	High	Pain, redness, decreased vision				Acute hypertensive uveitis
19 [26]	24 F	4				+	Local panuveitis
20 [27]	62 M	>50	Decreased vision, redness and pain of the eye			+	
21 [28]	41 F	44	Redness of the eye, pain, headache	+	+	+	Uveitis anterior secondary glaucoma
22 [29]	67 M	35	Pain, blurred vision increasing develeoped over 9 days	+	+	+	
23 [present case]	62 F	48	Pain, Redness, Blurred vision	+		+	Iritis with steril hypopyon
24 [30]	88 F	47	Acute blurred vision, increasing pain, redness	+	-	+	
25 [31]	27 M	18	Redness, photophobia, decreased vision			+	Keratoconjunctivitis
26 [32]	28 M	50	Redness, pain 5 days prior			+	Uveitis
27 [33]	70 M	26	Declined vision			+	Panuveitis

**Table 2:** Overview of general findings in reviewed cases

	27 patient in all	Data available
Generall findings:		
Age distribution	24–88	27/27
Gender	15 M/12 F	27/27
Immuno compromised	10	24/27
Diabetes mellitus	2	24/27
History of cancer	5	24/27
Flu like symtoms	7	15/27
Initial therapy:		In total
Only corticosteroide	12	22/27
Corticosteroids with additional antibiotics	8	
Diagnostic:		In total
Direct Gram stain	6 pos/9 neg	15/27
Aquous culture	18 pos/3 neg	21/27
Intraocular culture	7 pos/ 2 neg	9/27
Blood culture	3 pos/10 neg	13/27
Final therapy:		In total
Systemic (Intravenous, oral)	24/2	26/27
Ampicillin	15	
Gentamicin	7	
Penicilline	6	
Vancomycin	3	
Meropenem	2	
Erythromycin	1	
Tetracycline	1	
Ciprofloxacin	1	
Tobramycin	1	
Ceftazidime	1	
Clindamycin	1	
Combined	12	
Ocular additive		
Paraocular (subconjunctival/-tenon/ intraorbital)	5/1/1	
Intraocular (intracameral/ -vitreal)	2/8	
Time between onset and treament	4 to 32 days	24/27

*Listeria monocytogenes* is a catalase-positive and gram-positive rod that exhibits weak beta-haemolytic growth on blood agar. It is ubiquitously found and colonizes a wide variety of animals, soil, and vegetables. Transmission usually occurs via ingestion of contaminated food or by colonized gastrointestinal tract, in the case of mother to fetus transmission. Isolation of *Listeria monocytogenes* from non-sterile origins can be difficult. In history, for enhanced recovery, the cold enrichment procedure was done. Nowadays culturing of this pathogen is easily performed by standard media and lab procedures, in our case identification was performed by MALDI-TOF MS (matrix-assisted laser desorption & ionization time-of-flight mass spectrometry) analysis.

The cause of bacterial endophthalmitis was isolated by recovering *Listeria monocytogenes* from the anterior chamber (18/21; 86 %), vitreous fluid (7/9; 78 %), or in three cases from blood cultures (3/13; 23 %) (Table 2) [18, 22, 30].

The direct performed Gram stain from ocular samples was positive in only six (40 %) of fifteen published cases, which emphasizes the role of the microbiology culture with a higher sensitivity from ocular tissue (aqueous 18/21; 85 % and vitreous 7/9; 77 %) and blood cultures (3/13; 23 %). Nevertheless, the time period between the clinical presentation of the patient and a positive microbiological identification depends on selected samples, the bacterial load in the sample, prior administered anti-infective drugs, and techniques for identification of pathogens (e.g., biochemical profiling, PCR methods).

The time between the presentation of the patient and the beginning of an effective treatment against *Listeria monocytogenes* in the reviewed cases had a wide range between 4 and 32 days (mean 13.0 days, IQR = 12).

Endophthalmitis is a serious clinical problem with difficult management and often a very poor visual outcome [2, 5]. The final VA in patients with bacterial-caused endogenous endophthalmitis treated with the according antibiotics is in 70 % of all cases reduced to counting fingers or less [5]. The literature review by Jackson et al. found liver abscess (with *Klebsiella pneumoniea* as dominant species) to be the most common extraocular foci of infection, followed by pneumonia, endocarditis, soft tissue infection, urinary tract infection, meningitis, septic arthritis, and orbital cellulitis [5, 35]. Likely due to subtherapeutic levels of antibiotics within the eye, patients with known systemic infection may develop endophthalmitis [36].

The clinical course of endophthalmitis caused by *Listeria monocytogenes* had very different outcomes. In single cases, the infection led to enucleation, blindness, or strong visual loss [19, 30, 31, 33]. In eight cases (30 %), a cataract developed, which led to decreased VA with subsequent cataract surgery [16, 18, 22, 24, 25, 27–29]. Further ocular surgical operations were needed in five other cases (19 %); two patients needed a keratoplasty, and two other patients needed a trebeculectomy (7 % each) to manage the secondary glaucoma [13, 21, 22]. One patient required an additional vitrectomy with membrane peeling due to a macula hole and an irisprintlens because of permanent pupillary dilation [28].

**Fig. 3:**
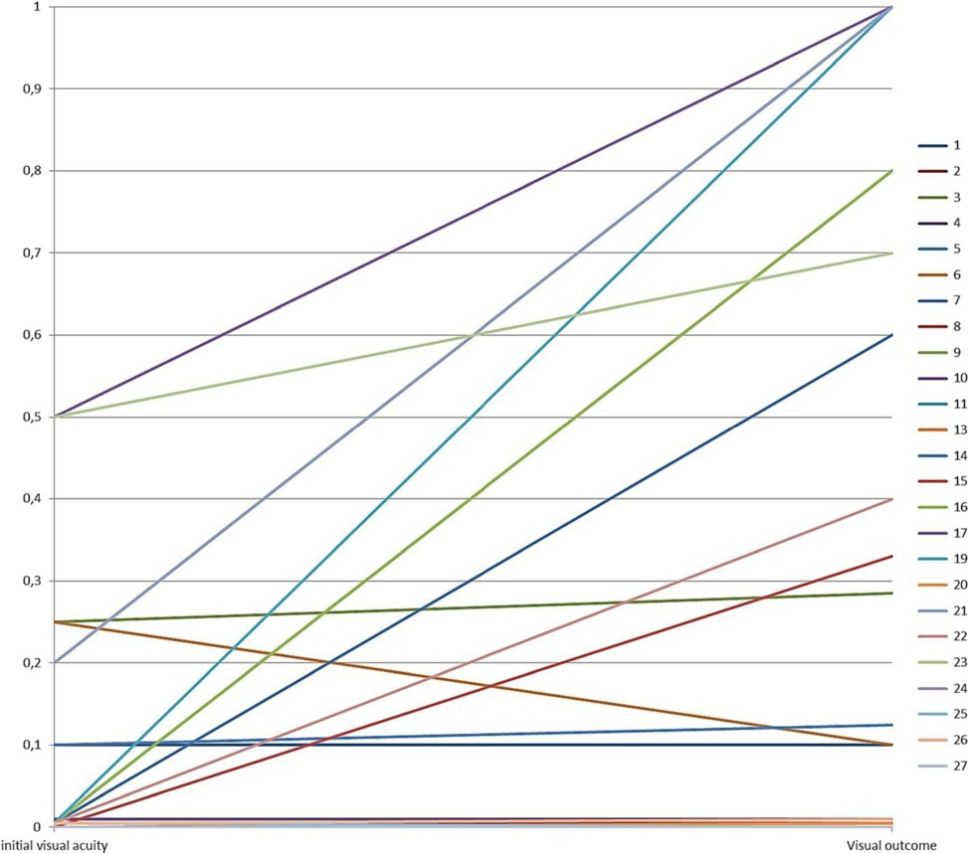
Visual acuity on admission and visual outcome. Visual acuity (VA) after stabilization of the ocular condition (visual outcome) was compared to VA at the time of admission to the hospital. For better graphical illustration, counting fingers (CF) and hand movements vision (HM) were converted into a decimal VA of 0.010 for CF and 0.0052 for HM according to the FrACT measures [42]. Since light perception could not be assigned to a specific decimal number, it was defined as 0 as well as no light perception. Case reports without description of either VA on admission or visual outcome were not considered [25, 34]

Patients with a high VA on admission show further improvement; in only one case, an initial VA of over 0.1 resulted in a decreased visual outcome. Even patients with initial very low VA of < 0.1 showed a tendency to recover and actually reach 1.0 VA. Overall, in seven cases, the VA had significantly improved to at least 0.6 or had completely recovered (Fig. 3), which can be explained by the main focus of inflammation in the anterior segment of the eye with little or late involvement of the retina [15, 17, 20, 23, 24, 26, 28]. After resolving of the hypopyon and clearing of the anterior chamber, VA increases. When the starting VA is below 0.05, the chance of improvement adds up to only 35.3 %. The remaining patients maintain low vision or VA decreases.

The main reasons for a poor visual outcome after endophthalimitis with *Listeria monocytogenes* are the late onset of diagnosis and, therefore, deferred treatment. Many patients are initially misdiagnosed due to the rare occurrence of this infection and also because the patients present with similar symptoms and ocular findings to sterile uveitis in the beginning of the disease (Table 1). Surprisingly, the initial treatment did not always have a high impact on visual outcome. In the group of patients treated with only corticosteroids at the onset of the disease, only five patients with an initial VA of 0.2 and HM even reached a visual outcome of 0.8 or 1.0. The remaining seven patients, on the other hand, did not reach a higher VA than 0.25. Unfortunately, the duration of sole corticosteroidal treatment could not be resembled in most cases. Whereas in the group of patients which were treated with corticosteroids and antibiotics, only two cases (including the present case) showed a visual outcome of higher than 0.4. Here, a more advanced stage of the disease on admission is assumed, which would explain the early beginning of antibiotic treatment and poor visual outcome, even though the initial diagnosis was still uveitis.

In the Endophthalmitis Population Study of Western Australia, Ng et al. observed noticeable changes in the diagnosis and management of postoperative endophthalmitis since 1995. They showed that the visual outcomes remain poor and have not improved, despite the fact that overall use of vitrectomy and intravitreal antibiotic have increased significantly, whereas the use of both subconjunctival and intravenous antibiotics have decreased [37]. Treatment depends on the underlying cause of endophthalmitis. When the focus is located extraocularly followed by an alteration in the blood ocular barriers (compared to exogenous endophthalmitis), systemic antibiotics can achieve therapeutic levels in the eye due to the disrupted blood ocular barrier and bactericidal intraocular antibiotic concentrations are realized [38]. The suggestion was made that systemic antibiotics are more valuable in endogenous endophthalmitis than in postoperative or traumatic endophthalmitis. Further, intraocular antibiotic injection and vitrectomy make only a limited contribution to successful treatment in endogenous infection [8]. On the other hand, different studies showed that eyes undergoing pars plana vitrectomy are three times more likely to retain useful vision. In addition they are three times less likely to require evisceration or enucleation due to satisfactory drug concentration in the vitreous by the intravitreal route [5, 39]. To prevent irreversible tissue destruction by the absence of adequate antimicrobial concentrations, intravitreal administration of antibiotics has become the main basis of endophthalmitis management [39–41]. In endogenous endophthalmitis the source of infection, despite of intense diagnostic investigations is often not found. Further patients with known systemic infection can develop endophthalmitis despite taking appropriate therapeutic systemic antibiotics [36]. *Listeria monocytogenes* has a high sensitivity to penicillin; in almost all cases, the intravenously applied antibiotic was either penicillin or ampicillin (Table 2). An early treatment with systemic antibiotic of dosage and frequency adequate to treat meningitis or other serious infections is suggested.

Since endogenous endophthalmitis is spread hematogenously, the choroids and ciliary body are usually the primary focuses of infection due to the higher blood flow. The retina and vitreous often show only secondary involvement [5]. In the case of infection with *Listeria monocytogenes*, the aqueous humor, followed by the vitreous, showed first signs of infiltration (Table 1). Although initially, in several cases as well as in the present case, the fundus could not be visualized, after vitrectomy or clearing of the anterior chamber no retinal involvement could be seen. Only one case described a retinal involvement which progressed to panophthalmitis and required enucleation. The further pathological investigation demonstrated an acute necrotizing panophthalmitis [33].

## Conclusion

*Listeria monocytogenes* is an uncommon cause of endopthalmitis. It is mostly spread endogenously by hematogenous dissemination, although a source of infection usually cannot be found. Typical clinical findings for endophthalimitis caused by *Listeria monocytogenes* include a massive fibrinous anterior chamber reaction with an increase of the intraocular pressure. The outcomes vary from benign reactions, such as mild uveitis, to severe reactions, like necrotising panophthalmitis. It is important to undertake appropriate micro sampling in order to diagnose atypical microorganisms (like *Listeria monocytogenes*), and initiate targeted antibiotic treatment, especially in patients who are immunocompromised. A prompt intervention is recommended in patients showing progressive eye symptoms.

## Abbreviations

CF, counting fingers; HM, hand movements vision; IQR, interquartile range; MALDI-TOF MS, matrix-assisted laser desorption & ionization time-of-flight mass spectrometry; sc, without refractive correction; VA, visual acuity
